# Development of a screen to identify selective small molecules active against patient-derived metastatic and chemoresistant breast cancer cells

**DOI:** 10.1186/bcr3452

**Published:** 2013-07-23

**Authors:** Keith M Gligorich, Rachel M Vaden, Dawne N Shelton, Guoying Wang, Cindy B Matsen, Ryan E Looper, Matthew S Sigman, Bryan E Welm

**Affiliations:** 1 Department of Oncological Sciences, Huntsman Cancer Institute, University of Utah, 2000 Circle of Hope Dr., Salt Lake City, Utah, 84112, USA; 2 Department of Chemistry, University of Utah, 315 S 1400 E, Salt Lake City, Utah, 84112, USA; 3 Department of Surgery, Huntsman Cancer Institute, University of Utah, 2000 Circle of Hope Dr., Salt Lake City, Utah, 84112, USA

## Abstract

**Introduction:**

High failure rates of new investigational drugs have impaired the development of breast cancer therapies. One challenge is that excellent activity in preclinical models, such as established cancer cell lines, does not always translate into improved clinical outcomes for patients. New preclinical models, which better replicate clinically-relevant attributes of cancer, such as chemoresistance, metastasis and cellular heterogeneity, may identify novel anti-cancer mechanisms and increase the success of drug development.

**Methods:**

Metastatic breast cancer cells were obtained from pleural effusions of consented patients whose disease had progressed. Normal primary human breast cells were collected from a reduction mammoplasty and immortalized with human telomerase. The patient-derived cells were characterized to determine their cellular heterogeneity and proliferation rate by flow cytometry, while dose response curves were performed for chemotherapies to assess resistance. A screen was developed to measure the differential activity of small molecules on the growth and survival of patient-derived normal breast and metastatic, chemoresistant tumor cells to identify selective anti-cancer compounds. Several hits were identified and validated in dose response assays. One compound, C-6, was further characterized for its effect on cell cycle and cell death in cancer cells.

**Results:**

Patient-derived cells were found to be more heterogeneous, with reduced proliferation rates and enhanced resistance to chemotherapy compared to established cell lines. A screen was subsequently developed that utilized both tumor and normal patient-derived cells. Several compounds were identified, which selectively targeted tumor cells, but not normal cells. Compound C-6 was found to inhibit proliferation and induce cell death in tumor cells via a caspase-independent mechanism.

**Conclusions:**

Short-term culture of patient-derived cells retained more clinically relevant features of breast cancer compared to established cell lines. The low proliferation rate and chemoresistance make patient-derived cells an excellent tool in preclinical drug development.

## Introduction

Over the last 40 years, advances in the development of breast cancer drugs have led to improved treatments and outcomes for patients [1,2]. However, mortality, which is generally attributed to metastatic disease and resistance to chemotherapy, has remained relatively unchanged over the same period [3,4]. In addition, many cancer drugs have significant toxicity, which impacts a patient's compliance with treatment and can result in serious long-term health effects [5]. These issues highlight the urgent need to develop new drugs that can target the chemoresistant disease while simultaneously reducing general toxicity to the patient.

Bringing a new investigational drug to the clinic is challenging and plagued by high failure rates [6,7]. Often, excellent efficacy in preclinical models does not translate into improved survival. One factor that may contribute to the high failure rate is a reliance on human preclinical models that do not accurately replicate clinical outcomes. For example, the most widely used *in vitro *model of breast cancer is established cell lines [8-10]. Even though cell lines share many molecular and genomic characteristics of breast cancer, their adaptation to culture can impart significant undesirable attributes that affect preclinical studies [11-13]. Compared to patient tumors, cell lines often exhibit increased proliferation, altered sensitivity to chemotherapy and reduced cellular heterogeneity [14-16]. Incorporation of new models that more accurately replicate features of cancer observed in patients, such as chemoresistance, metastasis and cellular heterogeneity, into drug development programs may lead to more successful clinical results for investigational therapeutics.

An alternative to established cell lines is the use of patient-derived tissue that is only briefly maintained in culture [8,14,17]. Short-term culture of patient-derived tissue is believed to retain many key features of the original tumor, including heterogeneity, proliferation rate and gene expression profiles [18]. In addition, tissue derived from patients previously treated with chemotherapy can acquire resistance through mechanisms developed naturally during the clinical course of therapy [19]. Therefore, incorporation of short-term cultures of patient-derived cells in drug screening assays is likely to identify compounds that circumvent chemoresistant pathways. Herein, we report the development of a drug screen to identify small molecules capable of selectively targeting chemoresistant patient-derived cancer cells.

## Methods

### Tissue culture and reagents

MCF-7 and MCF-10A cells were cultured with (D)MEM/F12 media with 2.5 mM L-glutamine and 15 mM HEPES buffer (HyClone, Logan, UT, USA) and the MDA-MB-231 and T47D cells were cultured with RPMI-1640 medium with 2.5 mM L-glutamine and 25 mM HEPES buffer (HyClone) at 37°C with 5% CO_2_. Both media were supplemented with 10% fetal bovine serum (heat inactivated, HyClone), 5.0 μg/mL of insulin-transferrin-selenium-X (ITS-X) (Life Technologies, Grand Island, NY, USA), penicillin-streptomycin-glutamine (Life Technologies), and 2.5 nM epidermal growth factor (EGF), recombinant human (BD Biosciences, San Jose, CA, USA).

De-identified pleural effusion (PE) and reduction mammoplasty tissue were collected by the Huntsman Cancer Institute Tissue Resource and Applications Core Facility with informed consent from patients at the Huntsman Cancer Hospital and the University of Utah Hospitals and Clinics under a protocol approved by the University of Utah Institutional Review Board [20]. Cells from freshly acquired effusion fluid were collected by centrifugation, washed with PBS and cryopreserved in 10% dimethyl sulfoxide (DMSO) (Sigma, St. Louis, MO, USA) and 90% human breast epithelial medium, which consists of (D)MEM/F12 supplemented with 15 mM HEPES, 5% fetal bovine serum (FBS), 1 mg/mL BSA (Sigma), 1 μg/mL ITS-X, 0.5 μg/mL hydrocortisone (Sigma), and 50 μg/mL gentamycin (Hyclone).

Tissue collected from a consented patient undergoing a voluntary reduction mammoplasty was digested with 2 mg/mL of collagenase (Sigma) and 100 U/mL of hyaluronidase (Sigma) at 37°C overnight to generate organoids. The organoids were cultured in modified M87 media, which consists of (D)MEM/F12 supplemented with 15 mM HEPES, 2% FBS, ITS-X, penicillin-streptomycin-glutamine, 5 ng/mL EGF, 0.3 μg/mL hydrocortisone, 0.5 ng/mL cholera toxin (Sigma), 5 nM 3,3',5-triiodo-L-thyronine (Sigma), 0.5 nM β-estradiol (Sigma), 5 μM (±)-isoproterenol hydrochloride (Sigma), 50 nM ethanolamine (Sigma) and 50 nM *O*-phosphorylethanolamine (Sigma) [21]. After two passages, the cells were immortalized utilizing a concentrated lentivirus that expresses the human telomerase gene under the control of the EF1α promoter at a multiplicity of infection (MOI) of 20. The immortalized cells, hTERT-HMEC, were subsequently expanded and early passages were cryopreserved in 10% DMSO and 90% modified M87 media. Both the hTERT-HMEC and patient-derived pleural effusion were cultured in modified M87 media at 37°C with 5% CO_2_. For each experiment requiring PE cells, only non-passaged, freshly defrosted cells were used following an 18-hour culture in modified M87 media. All hTERT-HMEC cells used were less than eight passages post immortalization, and primary PE cells were not cultured for longer than one week for any assay.

The following compounds were dissolved in DMSO (Sigma) and stored at -20°C: doxorubicin hydrochloride (Tocris Bioscience, Minneapolis, MN, USA), paclitaxel (taxol) (EMD Millipore, Billerica, MA, USA), gemcitabine hydrochloride (Sigma), 17-(allylamino)-17-demethoxygeldanamycin (17-AAG) (LC Laboratories, Woburn, MA, USA), bortezomib (LC Laboratories), panobinostat (LBH589) (Selleckchem, Houston, TX, USA), *cis*-diammineplatinum (II) dichloride (cisplatin) (Sigma), and staurosporine (Sigma). Chloroquine (Sigma) and tumor necrosis factor-related apoptosis inducing ligand (TRAIL), recombinant human (EMD Millipore) were dissolved in sterile water and were stored at -20°C. The small molecule C-6 was synthesized according to a previously published method [22], was dissolved in DMSO and stored at -20°C. For all experiments, the cells were seeded in their respective media and were allowed to recover overnight. Compounds were subsequently diluted in the corresponding media containing 2% FBS as well as a matched vehicle control, which did not exceed a final DMSO concentration of 0.2% (v/v).

### Cell characterization by flow cytometry

Non-confluent cultures of cell lines were trypsinized into single cell suspensions, washed with Hank's balanced salt solution (HBSS) supplemented with 2% FBS and counted. About 1.0 × 10^6 ^cells were incubated with fluorescently-conjugated antibodies for human CD24-fluorescein isothiocyanate (FITC) and CD44-allophycocyanin (APC) (BD Biosciences) on ice for 30 minutes. An additional 1.0 × 10^6 ^cells were stained with antibodies for human CD49f-FITC and EPCAM-APC (BD Biosciences) on ice for 30 minutes. Cell suspensions were subsequently washed with HBSS containing 2% FBS, resuspended at 3.0 × 10^6 ^cells/mL and were stained with 7-amino-actinomycin D (7-AAD) (BD Biosciences) on ice for 15 minutes. Cell suspensions were passed through a 70-μm cell strainer and were analyzed using a FACScan flow cytometer (BD Biosciences). The resulting data were analyzed with Flow Jo software (Tree Star, Ashland, OR, USA).

Patient-derived pleural effusion cells and hTERT-HMEC cells were cultured for two days in modified M87 media. Cells in suspension were collected and adherent cells were trypsinized and combined with the suspension cells. The resulting single cells were washed with HBSS containing 2% FBS and were counted. Two vials containing 1.0 × 10^6 ^cells were stained with a cocktail of phycoerythrin conjugated antibodies for human CD2, CD3, CD10, CD16, CD18, CD31, CD64 and CD140b (BD Biosciences) to exclude lineage positive cells (non-epithelial). Simultaneously, one vial was also stained with antibodies for human CD24-FITC and CD44-APC on ice for 30 minutes. The additional vial was stained with antibodies for human CD49f-FITC and EPCAM-APC (BD Biosciences) on ice for 30 minutes. Cell suspensions were subsequently washed with HBSS containing 2% FBS, resuspended at 3.0 × 10^6 ^cells/mL and were stained with 7-AAD on ice for 15 minutes. Cell suspensions were passed through a 70-μm cell strainer and were analyzed using a FACScan flow cytometer. The resulting data were analyzed with Flow Jo software.

### Dose response assays

Cells were seeded in white 96-well plates (Costar, Tewksbury, MA, USA) in 100 μL of their respective media at varying densities to achieve 80% to 90% confluency after five days in culture. The compounds dissolved in DMSO were diluted in their corresponding media containing 2% FBS and an EP Motion 5075 (Eppendorf North America, Hauppauge, NY, USA) liquid handler was utilized to perform a serial dilution. In addition, a vehicle control corresponding to the highest DMSO concentration, which did not exceed 0.2% (v/v), was also prepared. After the cells were cultured for 24 hours, the media were aspirated and the cells were treated with the compounds and vehicle controls in triplicate. After four days of treatment, viability was determined utilizing the ATPlite 1step assay system (Perkin Elmer, Waltham, MA, USA) according to the manufacturer's instructions. Luminescence measurements were acquired utilizing a Perkin Elmer 2104 EnVision plate reader. Raw luminescence values were normalized to the DMSO vehicle control wells. Normalized values were plotted as an average ± SD of three wells per concentration and these data were analyzed using the dose response nonlinear curve fitting function with Prism 5.0 (GraphPad Software, La Jolla, CA, USA) to determine the half maximal effective concentration (EC_50_).

### BrdU proliferation and cell cycle analysis

Cells were seeded in six-well plates (BD Falcon) in their respective media at different densities to attain 70% to 80% confluency at the end of the assay. In order to compare the proliferation rate of established cell lines compared to patient-derived cells, 10 μM of 5-bromo-2-deoxyuridine (BrdU) (Sigma) was added to the culture media for 30 minutes or 6 hours in triplicate. To determine the impact of C-6 on the cell cycle, cells were treated with 15 μM C-6 or 0.02% (v/v) DMSO vehicle in the corresponding media containing 2% FBS for 24 and 48 hours in triplicate followed by treatment with 10 μM BrdU for 30 minutes. Immediately following BrdU treatment, floating cells were collected and adherent cells were trypsinized. Floating and adherent cells were combined, washed with HBSS containing 2% FBS, fixed with 70% ethanol and stored overnight at -20°C. Cells were then treated with 2 M HCl containing 0.5% Triton X-100 (EMD Millipore) for 30 minutes at room temperature and were washed with 0.1 M sodium tetraborate (Sigma) at pH = 8.5. Next, cells were blocked with staining buffer, which consisted of 1% BSA, 0.5% Tween-20 (Fisher Scientific) in PBS, for five minutes and were stained with a mouse monoclonal BrdU antibody (Developmental Studies Hybridoma Bank, Iowa City, IA, USA) for one hour on ice. Cells were subsequently washed and stained with anti-mouse Alexa Fluor^® ^488 (Life Technologies) for 30 minutes on ice, washed, stained with 5 μg/mL of propidium iodide (EMD Millipore) and passed through a 70-μm cell strainer. Cell suspensions were analyzed using a FACScan flow cytometer and the resulting data were analyzed with Flow Jo software. The average ± SD of three wells for each condition was calculated.

### Measurement of proliferation by EdU incorporation

Cells were seeded in six-well plates and allowed to recover for 18 hours. Following the recovery time, 10 μM of 5-ethynyl-2'-deoxyuridine (EdU) (Molecular Probes) was added to the culture media of triplicate samples and the cells were cultured for 30 minutes or six hours. EdU incorporation was then quantified by flow cytometry using the Click-iT EdU Alexa Fluor 647 flow cytometry assay kit (Molecular Probes). The average ± SD of three wells for each condition was calculated.

### Characterization of mammary epithelial cell lineage markers

Non-passaged patient-derived plural effusion cells were defrosted and washed two times with HBSS. Cells were either stained immediately for mammary epithelial cell lineage markers or cultured for 96 hours and then stained. For staining, cytospin slides were prepared using 125,000 cells per slide (900 RPM, 10 minutes, Thermo Scientific Cytospin 4). The cells were fixed in 4% paraformaldehyde, permeabilized with 0.2% Triton X-100, and blocked with 1% BSA in PBS. The cells were then stained with antibodies for cytokeratin-8 (Developmental Studies Hybridoma Bank) and cytokeratin-14 (Covance). Following incubation with the appropriate secondary antibody (Alexa Fluor-conjugated IgG, Life Technologies) and 4',6-diamidino-2-phenylindole (DAPI), the slides were imaged using an IX81 microscope (Olympus, Center Valley, PA, USA).

### *In vivo *assessment of tumorigenicity

Three-week old female non-obese/severe combined immunodeficiency (NOD/SCID) mice were obtained from Jackson Labs (Bar Harbor, ME, USA) and maintained in a pathogen-free animal facility. All procedures were carried out in accordance with University of Utah-approved Institutional Animal Care and Use Committee protocols. The method used to assess the transformation capacity of primary reduction mammoplasty cells was performed similarly to those previously described [23]. Human mammary epithelial cells (HMEC) cells were infected individually or in combination with lentiviruses containing the human telomerase reverse transcriptase (hTERT) gene, the large T antigen of simian virus 40 (SV40-LT) gene and a constitutively active form of the human Ras (RasV12) gene. All genes were driven by the ubiquitously expressed EF1-α promoter. Cells were cultured in a supplemented media (modified M87) either in monolayer or in suspension. The day of the transplant, cells were washed and resuspended in Matrigel. The number 4 inguinal fat pad was cleared on one side and each recipient mouse received a 10 μL injection of approximately 750,000 cells suspended in Matrigel. At a minimum of forty-eight days post surgery, the transplanted glands were resected and fixed in 4% paraformaldehyde. The fixed tissue was then stained with carmine alum (Sigma) for 24 hours. Following one wash each in 70%, 95% and 100% ethanol, the gland was examined for the presence of tumors.

### Chemical screen

The hTERT-HMEC and PE1007070 cells were seeded in white 96-well plates (Costar) in 100 μL of modified M87 media at varying densities to achieve 80% to 90% confluency at the end of the assay. The 10 mM DMSO stock solutions from the 560-compound University of Utah Department of Chemistry library were diluted with modified M87 media utilizing an EP Motion 5075 (Eppendorf North America) liquid handler. In addition, corresponding DMSO vehicle and doxorubicin controls were prepared in modified M87 media. After the cells were cultured for 24 hours, 80 μL of media was aspirated from each well. Immediately, either 130 μL of the diluted compounds, DMSO vehicle or doxorubicin controls were added to each well in duplicate to achieve a final concentration of 20 μM for each compound with a DMSO concentration of 0.2% (v/v). After four days of treatment, viability was determined utilizing the ATPlite 1step assay system (Perkin Elmer) according to the manufacturer's instructions. Luminescence measurements were acquired utilizing a Perkin Elmer 2104 EnVision plate reader. Raw luminescence values were normalized to the DMSO vehicle control wells for each plate and cell type. Normalized average values for the PE1007070 cells were subtracted from the hTERT-HMEC cells to determine the selectivity for each compound. In addition, the normalized 20 μM doxorubicin and DMSO values were used to calculate the Z'-factor for each plate [24]. Additional information about the screen can be found in Additional file 1, Table S1.

### Live/dead assay

The hTERT-HMEC and PE1007070 cells were seeded in black wall clear bottom 96-well plates (Greiner Bio-one, Monroe, NC, USA) in 100 μL of modified M87 media at varying densities to achieve 80% to 90% confluency at the end of the assay. After 24 hours, the media were aspirated and the cells were treated with 20 μM C-6 or the corresponding 0.02% (v/v) DMSO vehicle control. After culturing for five days, the viability and cytotoxicity was determined with the Live/dead assay (Life Technologies) according to the manufacturer's instructions. Briefly, the media were aspirated and the cells were treated with 4 μM Calcein-AM and 8 μM Ethidium homodimer-1 diluted in (D)MEM/F12 for 30 minutes at 37°C. Subsequent imaging was performed with an IX81 microscope (Olympus, Center Valley, PA, USA) running Slide Book 5.0 software (Intelligent Imaging Innovations, Denver, CO, USA).

For the three dimensional culture experiments, PE904557a, PE900642a and PE11000025 cells were seeded in a 24-well ultra low adhesion plate (Costar) at approximately 2 × 10^6 ^cells/mL in mammary epithelial cell growth medium (MEGM) complete media (Lonza, Basel Switzerland) and were cultured overnight. The resulting aggregates were separated from single cells by differential centrifugation. Next, 20 μL of growth factor reduced Matrigel (BD Biosciences) was added to each well of a black walled clear bottom 24-well plate (Greiner Bio-one) and was allowed to solidify to form a base layer. The aggregates were then suspended in Matrigel on ice and approximately 300 aggregates in 40 μL were added to each well. After the Matrigel solidified, modified M87 media was added to each well and the cells were cultured overnight. The media were subsequently aspirated and the cells were treated with 30 or 60 μM C-6 or the corresponding 0.06% (v/v) DMSO vehicle control. After culturing for five days, the viability and cytotoxicity were determined utilizing the Live/dead assay (Life Technologies) in the same manner as described above. Multiple Z-planes of the organoids were subsequently imaged utilizing an IX81 microscope (Olympus) running Slide Book 5.0 software (Intelligent Imaging Innovations).

### AAF-Glo and caspase-Glo assays

Adherent cells were seeded in two identical white 96-well plates (Costar) in 100 μL of their respective media at varying densities to achieve 80% to 90% confluency at the end of the assay. After 24 hours, the media were aspirated and the cells were treated with 30 μM C-6 or a matched 0.03% (v/v) DMSO vehicle control in the corresponding media containing 2% FBS. For suspension cultures, cells were first seeded in 75 μL of media. At the start of treatment, 25 μL of a 4× concentration of C-6 or a matched (v/v) DMSO vehicle control was added to each well such that the final volume in each well was 100 μL and the final concentration of C-6 was 30 μM. For the caspase activity assays, additional wells were treated with 1 μM staurosporine. After culturing for the appropriate amount of time, either the AAF-Glo or Caspase-Glo assays (Promega, Madison, WI, USA) were performed on one 96-well plate according to the manufacturer's instructions. For the Caspase-Glo assay, cell viability was determined for the additional 96-well plate utilizing the ATPlite 1step assay system (Perkin Elmer) according to the manufacturer's instructions. For the AAF-Glo assay, the total protein in each well of the additional plate was measured using the BCA protein assay kit (Peirce, Rockford, IL, USA). Luminescence measurements were acquired utilizing a Perkin Elmer 2104 EnVision plate reader. Raw luminescence values for each assay were normalized to the DMSO vehicle control wells. The values for the Caspase-Glo or AAF-Glo assays were then normalized to the ATPlite values or total protein, respectively, in order to account for differences in cell numbers and were plotted as an average ± SD of four wells per condition.

### Western blot analysis

Cells were lysed in cold radioimmunoprecipitation assay buffer (50 mM Tris HCl, 150 mM NaCl, 0.1% sodium dodecyl sulfate (SDS), 0.5% sodium deoxycholate, 1% Triton X-100, pH = 8.0) supplemented with protease inhibitor cocktail (Sigma), phosphatase inhibitor cocktail 2 (Sigma) and 1 mM dithiothreitol (DTT) (Sigma). The lysate was sonicated for 30 seconds using a 450 Sonifier (Branson Ultrasonics, Danbury, CT, USA) and was centrifuged at 14,000 RPM for five minutes at 4°C. The protein concentration was determined using the BCA protein assay kit (Peirce, Rockford, IL, USA) and the samples were boiled for five minutes with 4× SDS Laemmli buffer. Equal amounts of protein were resolved on SDS polyacrylamide gels and transferred to an Immobilon-FL PVDF membrane (EMD Millipore). Blots were blocked in Odyssey Blocking Buffer (LI-COR, Lincoln, NE, USA) for one hour at room temperature, stained with primary antibodies overnight at 4°C, washed, and stained with IR800CW or IR680 anti-mouse or rabbit secondary antibodies (LI-COR) for one hour at room temperature. Blots were imaged with the Odyssey Infrared Imaging System (LI-COR). The following primary antibodies were purchased from Cell Signaling (Danvers, MA, USA): caspase-8, cleaved caspase-9, PARP and LC3A/B. In addition, active caspase-3 antibody was purchased from BD Biosciences and both vinculin and α-tubulin antibodies were obtained from Sigma.

### Statistics

An unpaired students t-test using Welch's correction was performed using Graph Pad Prism 5.0 and *P *< 0.05 between groups was considered significant.

## Results

### Patient-derived cells replicate the cellular heterogeneity, proliferation rate and chemo-sensitivity of normal and cancer tissue

We sought to utilize a novel therapeutic screen to identify compounds that selectively target patient-derived chemoresistant breast cancer cells while exhibiting limited toxicity to normal human breast tissue. For the screen, tumor cells were isolated from patients who presented with a pleural effusion (PE), which is a buildup of fluid and metastatic breast cancer cells in their pleural cavity. We obtained 1.0 × 10^9 ^cells from a patient (PE1007070) who was initially diagnosed with an ER- PR- HER2- primary tumor, and 2.0 × 10^8 ^viable cells from a patient (PE1008032) who had an ER+ PR+ HER2+ tumor. The PEs were collected after both patients relapsed following numerous rounds of chemotherapy (additional information can be found in Additional file 2, Table S2). In addition, we generated a non-transformed primary mammary epithelial control cell line to determine the general toxicity of each compound. Viable primary HMECs were isolated from a 25-year-old patient undergoing a reduction mammoplasty who had no known family history of breast cancer. The HMECs were immortalized with a lentivirus expressing the human telomerase gene (hTERT) under the control of the ubiquitous EF1α promoter to generate a low passage human hTERT-HMEC control cell line [21], which did not form tumors when transplanted orthotopically into NOD/SCID mice [see Additional file 3, Table S3].

**Figure 1:**
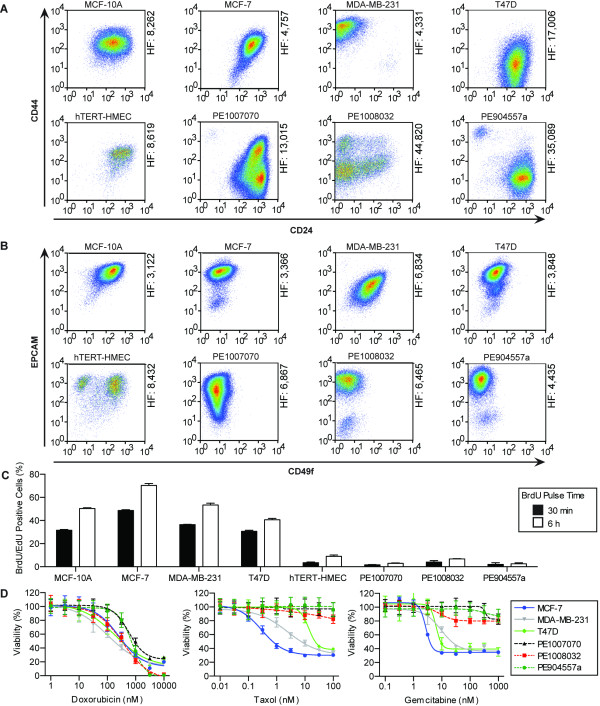
**Comparison of established cell lines and patient-derived cells**. **(A) **MCF-10A, MCF-7, MDA-MB-231, T47D, hTERT-HMEC, PE1007070, PE1008032 and PE904557a cells were stained with 7-AAD and a lineage cocktail in combination with CD44/CD24 or **(B) **CD49f/EPCAM and were analyzed by FACS. The heterogeneity factor (HF) (to the right of each graph) was calculated by multiplying the percent CV of each axis. **(C) **MCF-10A, MCF-7, MDA-MB-231, T47D, hTERT-HMEC, PE1007070, PE1008032 and PE904557a cells were treated with 10 μM BrdU or EdU for either 30 minutes or six hours and then BrdU/EdU incorporation was analyzed by flow cytometry. The graph shows the percent BrdU/EdU positive cells. **(D) **Dose response curves of doxorubicin, taxol and gemcitabine against MCF-7, MDA-MB-231, T47D, PE1007070, PE1008032 and PE904557a cells after four days of treatment. Cell viability was measured using a luciferase-based ATP assay and was normalized to the vehicle control. Error bars represent standard deviation. 7-AAD, 7-amino-actinomycin D; BrdU, 5-bromo-2-deoxyuridine; CV, coefficient of variance; EdU, 5-ethynyl-2'-deoxyuridine; FACS, fluorescence activated cell sorting; human mammary epithelial cells; hTERT, human telomerase; PE, pleural effusion.

We next characterized the cellular heterogeneity of PE cells by both immunofluorescence and fluorescence activated cell sorting (FACS) of cell surface proteins. Immunofluorescence of PE tumor cells derived either directly from the patient or after culturing for 96 hours demonstrated the presence of both luminal (keratin-8 positive) and basal (keratin-14 positive) cell types [see Additional file 4, Figure S1]. In addition, cells were analyzed by FACS for cell surface proteins which differentiate luminal versus basal/myoepithelial cells (EPCAM/CD49f)[25] and cancer cells with tumor initiating capabilities (CD44/CD24) [16]. These data demonstrated that cells in the non-tumorigenic mammary epithelial cell line, MCF-10A, formed a single broadly dispersed population, which clustered by both CD44/CD24 and CD49f/EPCAM staining (Figure 1A-B). In contrast, two distinct populations were observed in hTERT-HMECs stained with CD49f/EPCAM antibodies, which indicated the presence of both luminal and basal/myoepithelial cells [25].

To further assess the heterogeneity of the different cell types, the percent coefficient of variance (% CV = SD/mean * 100) was calculated from the histogram for each stain. A heterogeneity factor was subsequently calculated by multiplying the CV for each axis. The heterogeneity factor for CD49f/EPCAM was larger for the hTERT-HMEC compared to the MCF-10A cells, which suggests HMEC cells are more heterogeneous in regard to luminal and myoepithelial cell populations. In addition, CD24/CD44 staining of the established tumor cell lines MCF-7 (Luminal ER+, PR+, HER2-), T47D (Luminal ER+, PR+, HER2-) and MDA-MB-231 (Basal ER-, PR-, HER2-) indicated these cells had one main population (Figure 1A-B and Additional file 5, Figure S2). In contrast, PE1007070, PE1008032 and PE904557a (ER-, PR-, HER2+) cells contained several populations, including a CD44^Hi^/CD24^Low ^population reported to have enhanced tumor-initiating capacity [16]. Additionally, the heterogeneity factors for CD24/CD44 were higher for the PE1007070, PE1008032 and PE904557a compared to the MCF-7 and MDA-MB-231 cells. Together, these data illustrate that the hTERT-HMEC and patient-derived PE tumor samples are more heterogeneous compared to established cell lines.

The process of establishing cell lines is likely to impose selective pressure that favors highly proliferative cell populations [8]. Therefore, we wanted to compare the proliferation rates of cell lines and patient-derived primary tumor cells. For this study, established cell lines and patient-derived cells were treated with BrdU or EdU, for either 30 minutes or 6 hours, and then analyzed by flow cytometry (Figure 1C). Established cell lines were found to have BrdU/EdU incorporation ranging from approximately 30% to 50% and 50% to 70% when treated for 30 minutes and 60 minutes, respectively. In contrast, patient-derived cells had significantly lower BrdU/EdU incorporation, ranging between 0.4% to 7%. Importantly, the proliferation rate observed in patient-derived cells was similar to the 3.2% median BrdU incorporation measured in tumors removed from breast cancer patients treated with BrdU prior to surgery [26]. These data demonstrate widely disparate proliferation rates between established cancer cells lines and patient tumors. Furthermore, short-term culture of patient-derived tissue more closely matched the lower proliferation rate observed in patients.

Since PE tumor cells were isolated from patients with therapeutically recalcitrant disease, we wanted to determine if these samples were more resistant to chemotherapies used in the treatment of metastatic breast cancer [27]. To address this, we performed a four-day dose response experiment comparing the efficacy of different drugs against established cell lines and patient-derived cells (Figure 1D, Additional file 6, Table S4, and Additional file 7, Figure S3). We observed that doxorubicin, a chemotherapy that inhibits topoisomerase II [1], reduced the viability of the established tumor cell lines and patient-derived PE cells in a similar manner. In contrast, taxol, a microtubule inhibitor [28], and gemcitabine, a fluorinated pyrimidine [29], which both target rapidly dividing cells, significantly reduced the viability of established cell lines, but not the slowly dividing PE cells. In general, these dose response experiments indicated that patient-derived cells are more resistant to anti-proliferative chemotherapy than established cell lines, which correlates with the difference in mitotic rates between these cells. Together these experiments suggest that the patient-derived cells are resistant to several chemotherapies used in the treatment of metastatic breast cancer.

### Screen for compounds that selectively kill patient-derived metastatic cancer cells

**Figure 2:**
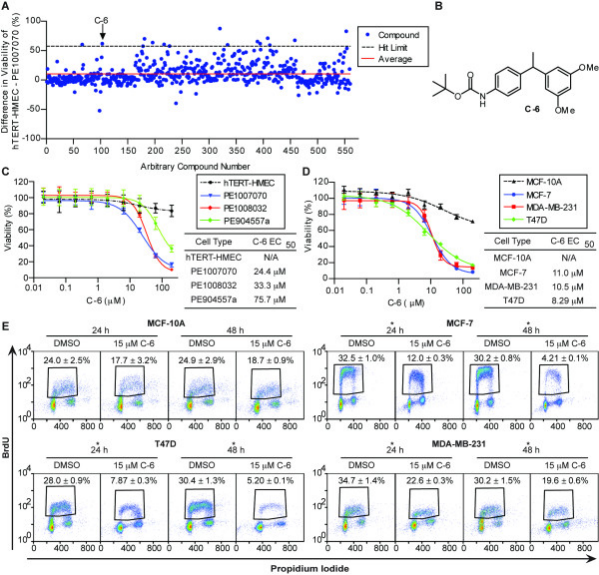
**Identification of the small molecule C-6**. **(A) **PE1007070 and hTERT-HMEC cells were treated with 20 μM of the compound library in duplicate. After four days, a luciferase-based ATP assay was performed and data were normalized to DMSO vehicle control wells to determine viability (%). The difference in viability between the hTERT-HMEC and PE1007070 cells is plotted. The hit limit was 2.7 times the standard deviation. **(B) **The structure of C-6, which had a 62% difference in viability between the hTERT-HMEC and PE1007070 cells. **(C) **Dose response curves of C-6 and EC_50 _values of hTERT-HMEC, PE1007070, PE1008032, and PE904557a cells and **(D) **MCF-10A, MCF-7, T47D and MDA-MB-231 cells after four days of treatment. N/A indicates data could not be fitted. **(E) **MCF-10A, MCF-7, MDA-MB-231 and T47D cells were treated with DMSO or 15 μM C-6 for 24 or 48 hours followed by addition of 10 μM BrdU for 30 minutes. The cells were stained for BrdU and propidium iodide and analyzed by flow cytometry. The average ± standard deviation of the percent BrdU positive cells (S phase) of three replicates was calculated. Asterisks (*) denote *P*-value < 0.05. BrdU, 5-bromo-2-deoxyuridine; DMSO, dimethyl sulfoxide; HMEC, human mammary epithelial cells; hTERT, human telomerase; PE pleural effusion.

Due to their clinically important features, such as low proliferation rates, chemoresistance and cellular heterogeneity, we reasoned that patient-derived tumor cells would be well-suited to identify novel anti-cancer compounds. Therefore, we performed a pilot screen utilizing patient-derived tumor cells and hTERT-HMECs to identify compounds that selectively reduced the viability of cancer cells without causing general cytotoxicity against normal breast cells [see Additional file 1, Table S1]. A 560-compound library was generated at the University of Utah Department of Chemistry that contains numerous vetted natural product pharmacophores for anti-cancer and antibiotic applications. The library was evaluated in duplicate at 20 μM against both hTERT-HMECs and patient-derived PE1007070 cells. After four days of treatment, a luciferase-based ATP assay was performed to assess viability and the average value for each compound was normalized to the DMSO vehicle control. For each compound, the percent viability of PE1007070 cells was subtracted from the viability of hTERT-HMECs to determine a compound's percent selectivity (Figure 2A). The average anti-cancer selectivity was calculated to be 10.2% and the hit limit was set at 2.7 times the SD, which led to a hit rate of approximately 3% or 15 compounds. In addition, 20 μM doxorubicin served as a positive control for each plate and was used to calculate the Z'-factor [24]. The Z'-factor for the DMSO and doxorubicin control was found to be >0.5, which is considered an excellent assay [see Additional file 8, Figure S4].

We performed a follow-up dose response experiment with hTERT-HMECs and PE1007070 cells to further validate the selectivity of 14 hits identified in the screen [see Additional file 9, Figure S5A and S5B]. About 50% of the original hits exhibited selectivity for patient-derived tumor cells compared to the hTERT-HMECs. In order to provide additional validation of the drug screen, we further evaluated hit 6, which was termed C-6 (Figure 2B), because it exhibited excellent selectivity for tumor cells and can be readily synthesized utilizing a unique palladium-catalyzed reductive coupling reaction[22]. Thus, additional dose response experiments of C-6 were performed on PE cells representing the three major subtypes of breast cancer including ER+/PR+/HER2- (PE1008032), ER-/PR-/HER2- triple negative (PE1007070), and ER-/PR-/HER+ (PE904557a) (Figure 2C). Whereas treatment with C-6 resulted in EC_50 _values in the range of 20 to 30 μM in both the ER+ and triple negative PEs, the HER2+ tumor exhibited a higher EC_50 _of 75.7 μM, which may suggest some resistance in HER2+ tumors. Importantly, C-6 did not significantly reduce the viability of the hTERT-HMECs. These results demonstrated that C-6 has exceptional selectivity for primary tumor cells compared to normal hTERT-HMECs and provides additional validation of the screening methodology.

After identifying the novel small molecule C-6, we wanted to investigate the compound's mechanism of action. Since patient-derived tumor cells are a limited resource, we needed to determine if established cell lines could be employed for mechanism-of-action studies. A dose response experiment of C-6 was performed on several established cell lines to determine the efficacy of C-6 (Figure 2D). An EC_50 _of 11.0 μM was measured for MCF-7 cells, 10.5 μM for MDA-MB-231cells and 8.29 μM for T47D cells, which suggests that C-6 has slightly higher activity against the established cell lines compared to the patient-derived cells. Importantly, more than 70% of untransformed MCF-10A cells were still viable even with 200 μM C-6 treatment which further supports a cancer selective mechanism-of-action.

To begin to elucidate C-6's cancer-selective mechanism of action, we performed experiments to assess the effects of this compound on proliferation and cell death. Cell cycle analysis was performed using cell lines due to the low baseline proliferation rate in PE cells. To study C-6's impact on the cell cycle, MCF-10A, MCF-7, MDA-MB-231 and T47D cells were treated with DMSO or 15 μM C-6 for 24 or 48 hours and were incubated with BrdU for 30 minutes followed by FACS analysis (Figure 2E). Interestingly, treatment with C-6 induced a significant reduction in the percent of BrdU positive cells (S phase) and increased the percentage of cells in G_1_/G_0 _in each cancer cell line [see Additional file 10, Figure S6]. In contrast, the untransformed MCF-10A cells did not show a statistically significant difference in their cell cycle profile. Together these data demonstrate that C-6 causes a selective cytostatic phenotype in breast cancer cell lines.

### C-6 selectively induces a caspase-independent cell death mechanism

**Figure 3:**
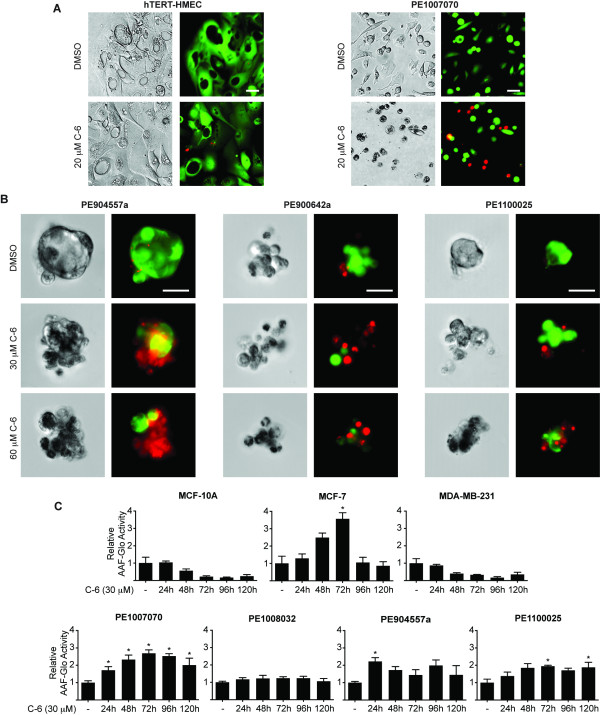
**C-6 can induce death in cancer cells**. **(A) **Differential interference contrast (DIC), calcein-AM fluorescence (green), and ethidium homodimer-1 (red) images of hTERT-HMEC and PE1007070 cells cultured in two dimensions and treated with DMSO or 20 μM C-6 for five days (Scale bar is 50 μm). **(B) **Confocal DIC, calcein-AM fluorescence (green), and ethidium homodimer-1 (red) images of PE904557a, PE900642a, and PE1100025 cells cultured in three-dimensional Matrigel and treated with DMSO or either 30 or 60 μM C-6 for five days (Scale bar is 50 μm). **(C) **MCF-10A, MCF-7, MDA-MB-231, PE1007070, PE1008032, PE904557a, and PE1100025 cells were treated with DMSO or 30 μM C-6 for 24 to 120 hours. The relative amount of released protease activity was measured using a luciferase-based AAF-Glo assay and these data were normalized to total protein measured with a BCA assay. Asterisks (*) denote *P*-value < 0.05. DMSO, dimethyl sulfoxide; HMEC, human mammary epithelial cells; hTERT, human telomerase; PE, pleural effusion.

Since C-6 was found to cause a reduction in proliferation, we wanted to determine if the compound was also inducing cell death. Accordingly, hTERT-HMECs and PE1007070 cells were cultured in monolayer and treated with 20 μM C-6 and a live/dead assay was performed (Figure 3A). The compound did not induce a gross morphological phenotype in the hTERT-HMECs or a significant increase in dead (ethidium bromide positive) cells. In contrast, C-6 caused the PE1007070 cells to become rounded up and led to an increase in the number of dead cells compared to the DMSO vehicle control.

After determining that C-6 induced cell death in the PE1007070 cells cultured in monolayer, we wanted to investigate if the small molecule was also active against cells cultured in three-dimensions, which has been proposed to be a better model of breast cancer due to establishing cell-cell interactions similar to tumors *in vivo *[30-32]. Accordingly, PE904557a, PE900642a and PE1100025 cells were cultured overnight in ultra low adhesion plates to facilitate aggregation. The resulting aggregates were embedded in Matrigel and treated with DMSO or either 30 or 60 μM C-6 for five days (Figure 3B). The live/dead assay was performed and it was found that C-6 was able to induce cell death in patient-derived samples cultured in three-dimensions.

In order to quantify cell death more accurately, both established cell lines and primary PE cells were treated with DMSO or 30 μM C-6 and analyzed for proteases released from dying cells every 24 hours for five days using an AAF-Glo assay (Figure 3C). Treatment of MCF-10A cells with C-6 did not cause an increase in the relative AAF-Glo activity, which indicated that C-6 does not induce death in these cells. However, treatment of MCF-7 cells and PE cells from three different patients resulted in a significant increase in the relative AAF-Glo activity compared to DMSO vehicle-treated cells. Interestingly, MDA-MB-231 and PE1008032 cells, which where both highly sensitive to C-6 in dose response assays (Figure 2C and 2D), did not have increased AAF-Glo activity, which suggests that C-6's mechanism of action in these cells is cytostatic. These data demonstrate that C-6 can induce cell death and/or cytostatic effects in tumor cells, but not in untransformed breast cells.

**Figure 4:**
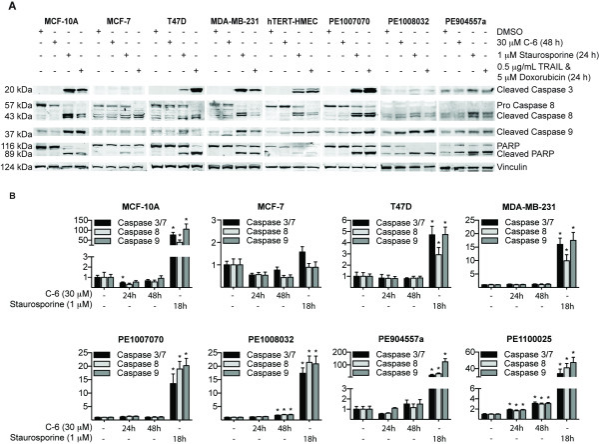
**C-6 does not induce caspase or PARP cleavage**. **(A) **MCF-10A, MCF-7, T47D, MDA-MB-231, hTERT-HMEC, PE1007070, PE1008032 and PE904557a cells were treated with DMSO (48 hours), 30 μM C-6 (48 hours), 1 μM staurosporine (24 hours) or 1 μg/mL TRAIL and 5 μM doxorubicin (24 hours) and the resulting whole cell lysates were analyzed by Western blot for cleaved caspase 3, caspase 8, cleaved caspase 9 and PARP. **(B) **MCF-10A, MCF-7, T47D, MDA-MB-231, PE1007070, PE1008032, PE904557a and PE110025 cells were treated with DMSO (48 hours), 30 μM C-6 (24 and 48 hours) or 1 μM staurosporine (24 hours) and the relative amount of caspase activity was measured using a luciferase-based Caspase-Glo assay and normalized to cell viability using a luciferase-based ATP assay. DMSO, dimethyl sulfoxide; HMEC, human mammary epithelial cells; hTERT, human telomerase; PE, pleural effusion; TRAIL, tumor necrosis factor related apoptosis inducing ligand.

We next wanted to investigate whether the death mechanism was mediated through caspase-induced apoptosis [33,34]. For this analysis, whole cell lysates derived from either DMSO or C-6 treated cells were analyzed by Western blot for cleaved caspase-3, caspase-8, cleaved caspase-9, and PARP (Figure 4A). In contrast to positive control compounds, C-6 did not induce cleavage of caspase-3, -8, -9, or PARP. A luminescence-based caspase activity assay was also performed to further confirm that C-6 was not activating caspase-3/7, -8 or -9 (Figure 4B). Treatment with 30 μM C-6 for 24 or 48 hours did not induce significant caspase activity. In addition, the pan-caspase inhibitor Z-VAD-FMK did not affect C-6 induced cell death in MCF-7 cells (data not shown). Taken together, these data demonstrate that C-6 can induce cell death through a caspase-independent mechanism.

We next evaluated if C-6 induces cell death through autophagy, which has been shown to occur in a caspase-independent manner [35,36]. During autophagy, the protein LC3A/B-I is processed into the lower molecular weight form LC3A/B-II [37], which can be detected by Western blot. Cell lines and PE cells were treated with DMSO, 30 μM C-6, 1 μM staurosporine or 50 μM chloroquine, a compound known to cause LC3-II accumulation [38]. The resulting whole cell lysates were analyzed by Western blot for the presence of LC3A/B-II [see Additional file 11, Figure S7]. While chloroquine led to a significant increase in LC3A/B-II levels in most cells, C-6 only induced a small increase in LC3A/B-II levels in the T47D cells, but not the other cell types evaluated. In addition, autophagosomes were not observed in MCF-7 cells by fluorescence microscopy using LC3-EGFP (data not shown). Taken together, these data demonstrate that C-6 induces caspase-independent, non-autophagic cell death selectively in cancer cells.

## Discussion

The data presented here show that short-term culture (<1 week) of non-passaged patient-derived tissue retains many clinically-relevant characteristics of advanced stage breast cancer, such as heterogeneous cell populations, low cell proliferation and resistance to chemotherapy (Figure 1) [11-13]. As such, the integration of patient-derived tissue into drug development programs may facilitate the identification of compounds that target slowly dividing, metastatic tumor cells, which are intrinsically less sensitive to anti-proliferative chemotherapy.

Effectively incorporating patient-derived tissue into drug screens is challenging due, in part, to constraints on obtaining viable surgical specimens and difficulties expanding primary cancer cells to quantities necessary for large-scale screening. Recently, Gupta *et al*. developed a method that overcame some of these limitations. They demonstrated that modified HMLER cells, which were experimentally-derived by transforming normal HMECs with hTERT, SV40 large-T antigen and hRas(V12), could be readily cultured and screened to identify anti-cancer compounds that selectively target cancer stem cells [39]. Here, by performing a pilot screen of 560 compounds we demonstrate that patient-derived tissue can be employed directly in drug discovery screen without passaging. To our knowledge, our screen is the first report to use non-passaged patient-derived breast cancer cells in parallel with low passaged primary immortalized HMECs to identify cancer selective small molecules. Our pilot screen bypassed the necessity of expansion *in vitro *by using viable cells collected from patients with metastatic breast cancer who developed pleural effusions [20]. As an example of the number of cells that can be acquired during routine care of patients, 1.0 × 10^9 ^viable cells were isolated from a single patient (PE1007070), which is sufficient to culture almost 700 96-well format plates at 15,000 cells/well [see Additional file 2, Table S2]. If necessary, further expansion of primary PE cells can be achieved *in vivo*. A recent report by DeRose *et al*. demonstrated that tissue derived from both solid tumors and pleural effusions can be expanded by direct engraftment into immunodeficient mice [40]. Even after multiple serial transplantations, the grafts maintain the original tumor's growth, pathology and metastatic potential, which enables *in vivo *expansion of patient-derived cells and reduces the potential for culture-derived artifacts. While large scale *in vivo *expansion of patient-derived tissue is labor-intensive and costly, the identification of anti-cancer compounds that target treatment-recalcitrant tumors with low proliferation rates may lead to the identification of novel targets and new treatments for patients with metastatic or advanced disease.

As with any screening platform, reproducibility as well as false positives and negatives are problems that can reduce their usefulness in drug discovery [41]. In our screen, we were able to achieve excellent Z'-factors, which suggests limited plate-to-plate variability [see Additional file 8, Figure S4] [24]. However, subsequent dose response experiments of hits identified in the screen suggested about a 40% to 50% false positive hit rate [see Additional file 9, Figure S5]. It is likely that this false positive rate is attributed to the poor solubility of several compounds in the library, which contained novel compounds that were not previously optimized for biological assays. As an example, the calculated cLogP values of the 14 compounds identified in the initial screen ranged from 3.1 to 5.5 (average 4.2). Thus, the use of small molecule libraries pre-selected for drug-like compounds may reduce the false positive rate.

Our screen led to the identification of at least six novel compounds that exhibited validated selectivity in a secondary dose-response assay [see Additional file 8, Figure S4]. Further studies were performed on hit C-6, which was highly selective for cancer cells in the primary screen. The dose response experiments demonstrated that C-6 had comparable activity and selectivity for cancer cells representing the major subtypes of breast cancer including ER+ (MCF-7, T47D and PE1008032 cells), triple negative (MDA-MB-231 and PE1007070 cells) and HER2+ (PE904557a cells) (Figure 2C and 2D). Cancer cells treated with C-6 exhibited varied effects, including cell death and reduced proliferation, which were either not apparent or significantly lower in normal breast cells. Importantly, we found that C-6 does not cause apoptosis through the caspase signaling cascade, which is a cell death pathway often defective in chemoresistant cancer cells (Figure 4A and 4B) [42-44].

While C-6 was highly selective in our *in vitro *assays, its lipophilic properties, poor solubility and high cLogP value (calculated 5.3) make *in vivo *evaluation challenging. As such, assessment of the selectivity of C-6 for cancer cells in mouse models, and evaluation of toxicities, pharmacokinetics, and metabolic profiles will require optimization of its structure to enhance its bioavailabilty. Taken together, this study describes a small molecule screen using primary metastatic tumor cells and demonstrates the *in vitro *cancer selectivity of the novel compound C-6.

## Conclusions

Our results demonstrate that non-passaged primary tumor cells are more heterogeneous, have reduced proliferation rates and exhibit resistance to chemotherapy compared to established cell lines. Since patient-derived cells optimally replicate tumor growth, survival and chemoresistance mechanisms acquired in patients during disease progression, their use in drug discovery may lead to novel cancer therapeutics that target advanced stages of breast cancer. One of the compounds identified in the screen, C-6, was found to selectively inhibit cell proliferation and induce cell death in several patient-derived tumor cells via a caspase-independent mechanism. Our data suggest that patient-derived cells are a valuable tool with high potential in the development of new cancer drugs.

## Abbreviations

7-AAD: 7-aminoactinomycin; APC: allophycocyanin; BrdU: 5-bromo-2-deoxyuridine; BA: bovine serum albumin; CV: coefficient of variance; (D)MEM: (Dulbecco's modified Eagle's medium; DMSO: dimethyl sulfoxide; EC_50_: half maximal effective concentration; EDU: 5-ethynyl-2'-deoxyuridine; EGF: epithelial growth factor; EPCAM: epithelial cell adhesion molecule; FACS: fluorescence activated cell sorting; FBS: fetal bovine serum; FITC: fluorescein isothiocyanate; HBSS: Hank's balanced salt solution; HMEC: human mammary epithelial cells; hTERT: human telomerase; ITS-X: insulin-transferrin-selenium-X; NOD/SCID: non-obese/severe combined immunodeficiency; PBS: phosphate-buffered saline; PE: pleural effusion; TRAIL: tumor necrosis factor related apoptosis inducing ligand.

## Competing interests

The authors declare that they have no competing interests.

## Authors' contributions

KMG, RMV, DNS, CBM and MSS performed experiments. GW and CBM procured the patient-derived tissue. REL and MSS compiled the chemical library. KMG, RMV, MSS and BEW analyzed data. KMG wrote the paper. RMV, MSS and BEW edited the paper. MSS and BEW supervised the project. All authors read and approved the final manuscript for publication.

## Supplementary Material

Additional file 1 **Supplemental table 1**. Description of the screen, protocol, and data analysis. Click here for file

Additional file 2 **Supplemental table 2**. Primary cells patient background and chemotherapy history. Click here for file

Additional file 3 **Supplemental table 3**. Tumorigenicity of immortalized hTERT-HMEC cells in NOD/SCID mice. Click here for file

Additional file 4 **Supplemental figure 1**. Patient-derived cells retain mammary epithelial cell lineage markers during *in vitro *culture. PE1007070, PE1008032, and PE904557a cells were stained for mammary epithelial cell markers cytokeratin 8 (K8) and cytokeratin 14 (K14) before *in vitro *culture and after 96 hours of *in vitro *culture. Nuclei are stained with DAPI. Scale bar is 10 μm. Click here for file

Additional file 5 **Supplemental figure 2**. Characterization of cells by flow cytometry. (**A**) hTERT-HMEC, (**B**) PE1008032 (**C**) PE1007070 and (**D**) PE904557a were analyzed by flow cytometry for FSC/SSC, 7-AAD and Lineage markers. Click here for file

Additional file 6 **Supplemental table 4**. EC_50 _values of chemotherapies after four days of treatment. Click here for file

Additional file 7 **Supplemental figure 3**. Chemosensitivity of established cell lines and patient-derived cells. Dose response curves of bortezomib, LBH589, cisplatin and 17-AAG against MCF-7, MDA-MB-231, T47D, PE1007070, PE1008032 and PE904557a cells after four days of treatment. Cell viability was determined using a luciferase-based ATP viability assay, which was normalized to the untreated vehicle control. Error bars represent the standard deviation of four replicates. Click here for file

Additional file 8 **Supplemental figure 4**. The Z'-Factor for each plate was calculated using the average percent viability of the 20 μM doxorubicin wells (positive control) and 0.2% v/v DMSO wells (negative control). Click here for file

Additional file 9 **Supplemental figure 5**. **(A) **Dose response of the top 14 selective hits from the screen against the hTERT-HMEC and PE1007070 cells after four days of treatment. Cell viability was determined using a luciferase-based ATP viability assay, which was normalized to the untreated vehicle control. Error bars represent standard deviation. N/A denotes that data could not be fitted. **(B) **Representative small molecules and substructures of hits identified from the screen. Click here for file

Additional file 10 **Supplemental figure 6**. MCF-10A, MCF-7, T47D, and MDA-MB-231 cells were treated with DMSO or 15 μM C-6 for 24 hours or 48 hours followed by addition of 10 μM BrdU for 30 minutes. The cells were stained for BrdU, PI and analyzed by FACS to determine the percentage of cells in the G_1_/G_0_, S, and G_2_/M phase. Asterisks (*) denote *P*-value < 0.05 of difference between percentages of cells in S phase. Click here for file

Additional file 11 **Supplemental figure 7**. C-6-induced cell death is independent of autophagy. MCF-10A, MCF-7, MDA-MB-231, T47D, hTERT-HMEC, PE1007070, PE108032 and PE904557a cells were treated with DMSO (48 hours), 30 μM C-6 (48 hours), 1 μM staurosporine (24 hours) or 50 μM chloroquine (24 hours) and resulting whole cell lysates were analyzed by Western blot for LC3A/B. Click here for file
