# Supplementary material for: Development of a screen to identify selective small molecules active against patient-derived metastatic and chemoresistant breast cancer cells
Source: Breast Cancer Res. 2013 Jul 23;15(4):R58. doi: 10.1186/bcr3452 (PMC4028696; doi:10.1186/bcr3452)
Supplement: Additional file 1 — Supplemental table 1. Description of the screen, protocol, and data analysis. [file bcr3452-S1.PDF]

Supplemental Table 1. Description of the screen, protocol, and data analysis.

Category	Parameter	Description
**Assay**	Type of assay	Cell-based
	Target	Selective reduction of primary pleural effusion breast cancer cells (PE1007070) compared to non- tumorigenic immortalized human mammary epithelial cells (hTERT-HMEC)
	Primary measurement	Total ATP (Viability)
	Key reagents	ATP Lite One Step (Perkin Elmer)
	Assay protocol	Seed cells and culture for 24h. Add additional media containing compounds or controls, culture for 4 days and perform ATP viability assay
**Library**	Library size	560 compounds
	Library composition	Focused library of small molecules containing vetted natural product pharmacophores for anti-cancer and anti-biotic applications
	Source	University of Utah Chemistry Department
**Screen**	Format	96 well plate
	Concentration(s) tested	20 μM (0.2% v/v DMSO)
	Plate controls	0.2% v/v DMSO and 20 μM Doxorubicin
	Reagent/ compound dispensing system	Eppendorf EP Motion 5075 (Compound dilution)
	Detection instrument and software	Luminescence was detected with a Perkin Elmer Envision 2104
	Assay validation/QC	20 μM Doxorubicin was used as a positive control and used to calculate Z' Factor
	Correction factors	Background subtraction of wells containing media and ATP Lite One step reagent (no cells)
	Normalization	The average value for duplicate wells for each compound was normalized to DMSO vehicle control wells to determine percent viability
	Additional comments	Selectivity of compounds was determined by subtracting the average viability of the PE1007070 from the hTERT-HMEC cells
**Post-HTS analysis**	Hit criteria	The average selectivity (hTERT-HMEC - PE1007070 viability) was calculated and any compound with selectivity greater than 2.7 times the standard deviation
	Hit rate	3%
	Additional assay(s)	Dose response against hTERT-HMEC and PE1007070 cells was performed
	Confirmation of hit purity and structure	By ^{1}H NMR or LC-MS
	Additional comments	An approximate 50% false positive rate was observed after additional assays were performed. Some hits may be due to luciferase inhibition or due to compound insolubility causing unequal distribution of the suspension.
