# Supplementary material for: Development of a screen to identify selective small molecules active against patient-derived metastatic and chemoresistant breast cancer cells
Source: Breast Cancer Res. 2013 Jul 23;15(4):R58. doi: 10.1186/bcr3452 (PMC4028696; doi:10.1186/bcr3452)
Supplement: Additional file 2 — Supplemental table 2. Primary cells patient background and chemotherapy history. [file bcr3452-S2.PDF]

Supplemental Table 2. Primary cells patient background and chemotherapy history

Cells	Age	Race	Source	Primary Tumor	Sample Size/ Viable Cells	Chemotherapy History
**hTERT-HMEC**	25	African American	Reduction Mammoplasty	No cancer history	320 g/ 6.0 x 10^7 cells	N/A
**PE1007070**	61	Caucasian	Pleural Effusion	ER- PR- HER2-	1.1 L/ 1.0 x 10^9 cells	Gemcitabine, Carboplatin, Doxorubicin, Taxol, Capecitabine,
**PE1008032**	53	Caucasian	Pleural Effusion	ER+ PR+ HER2-	3.1 L/ 2.1 x 10^8 cells	Gemcitabine, Carboplatin, Doxorubicin, Taxol, Fluorouracil, Cyclophosphamide
**PE904557a**	52	unknown	Pleural Effusion	ER- PR- HER2+	0.6 L/ 1.9 x 10^8 cells	Herceptin, Navelbine (Full history unavailable)
**PE900642a**	67	unknown	Pleural Effusion	ER- PR- HER2-	1 L/ 2.4 x 10^8 cells	Faslodex, Taxol (Full history unavailable)
**PE1100025**	55	Caucasian	Pleural Effusion	ER+ PR+ HER2+	3 L/ 1.8 x 10^8 cells	Herceptin, Gemcitabine, Carboplatin, MM-111, Doxorubicin, Taxol, Navelbine
