# Supplementary material for: Development of a screen to identify selective small molecules active against patient-derived metastatic and chemoresistant breast cancer cells
Source: Breast Cancer Res. 2013 Jul 23;15(4):R58. doi: 10.1186/bcr3452 (PMC4028696; doi:10.1186/bcr3452)
Supplement: Additional file 3 — Supplemental table 3. Tumorigenicity of immortalized hTERT-HMEC cells in NOD/SCID mice. [file bcr3452-S3.PDF]

Supplemental Table 3. Tumorigenicity of immortalized hTERT-HMEC cells in NOD/SCID mice

Genotype	Number of Tumors/Injections
hTERT	0/7
SV40-LT	0/2
hRas(V12)	0/2
hTERT, SV40-LT	0/1
hTERT, SV40-LT, hRas(V12)	6/7
