# Supplementary figures and images for: Development of a screen to identify selective small molecules active against patient-derived metastatic and chemoresistant breast cancer cells
Source: Breast Cancer Res. 2013 Jul 23;15(4):R58. doi: 10.1186/bcr3452 (PMC4028696; doi:10.1186/bcr3452)

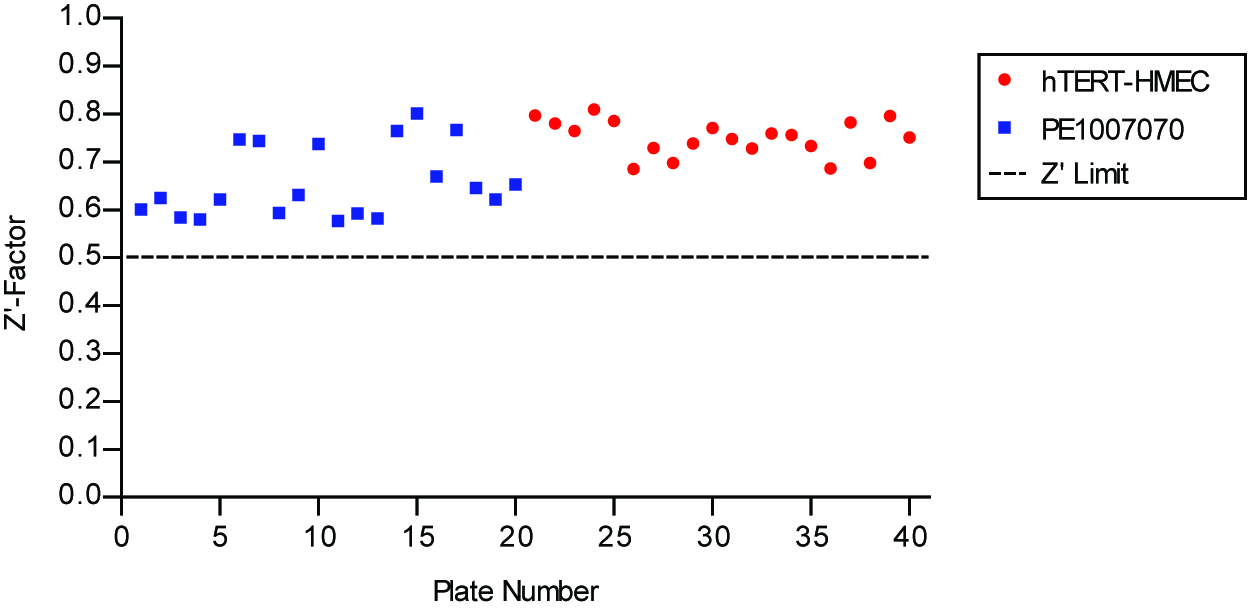

Supplement: Additional file 4 — Supplemental figure 1. Patient-derived cells retain mammary epithelial cell lineage markers during in vitro culture. PE1007070, PE1008032, and PE904557a cells were stained for mammary epithelial cell markers cytokeratin 8 (K8) and cytokeratin 14 (K14) before in vitro culture and after 96 hours of in vitro culture. Nuclei are stained with DAPI. Scale bar is 10 μm. [file bcr3452-S4.tif]

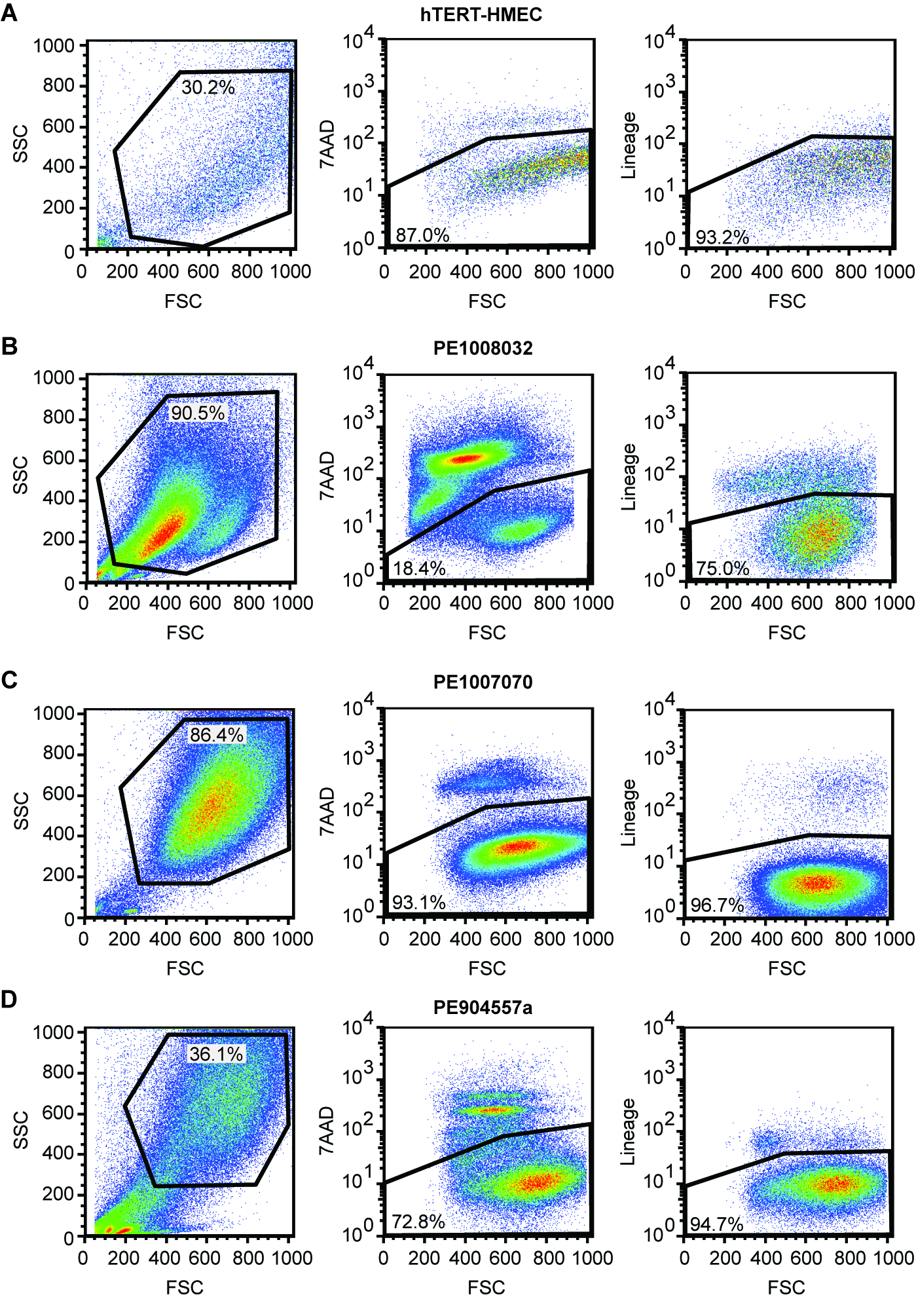

Supplement: Additional file 5 — Supplemental figure 2. Characterization of cells by flow cytometry. (A) hTERT-HMEC, (B) PE1008032 (C) PE1007070 and (D) PE904557a were analyzed by flow cytometry for FSC/SSC, 7-AAD and Lineage markers. [file bcr3452-S5.TIFF]

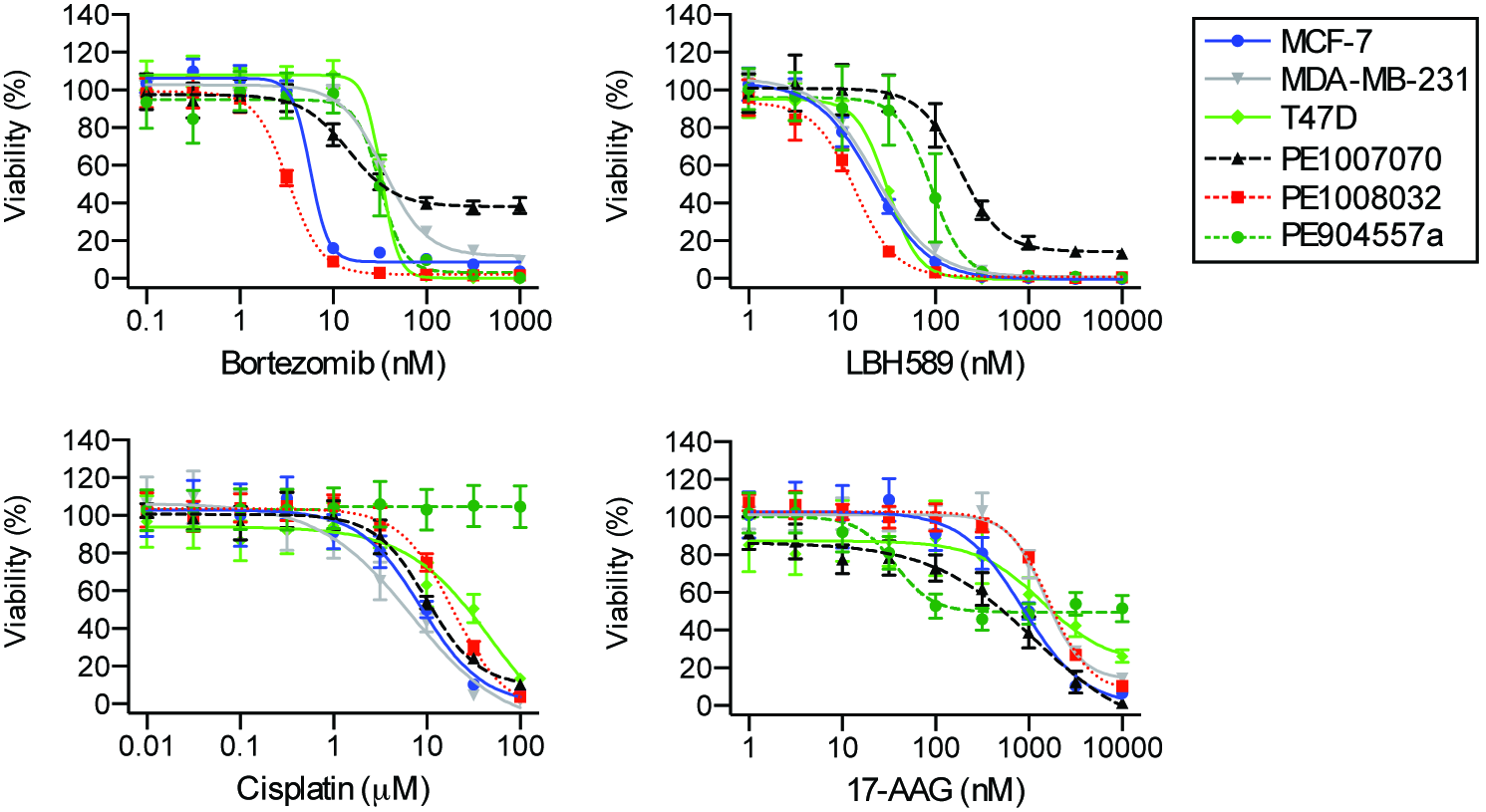

Supplement: Additional file 7 — Supplemental figure 3. Chemosensitivity of established cell lines and patient-derived cells. Dose response curves of bortezomib, LBH589, cisplatin and 17-AAG against MCF-7, MDA-MB-231, T47D, PE1007070, PE1008032 and PE904557a cells after four days of treatment. Cell viability was determined using a luciferase-based ATP viability assay, which was normalized to the untreated vehicle control. Error bars represent the standard deviation of four replicates. [file bcr3452-S7.TIFF]

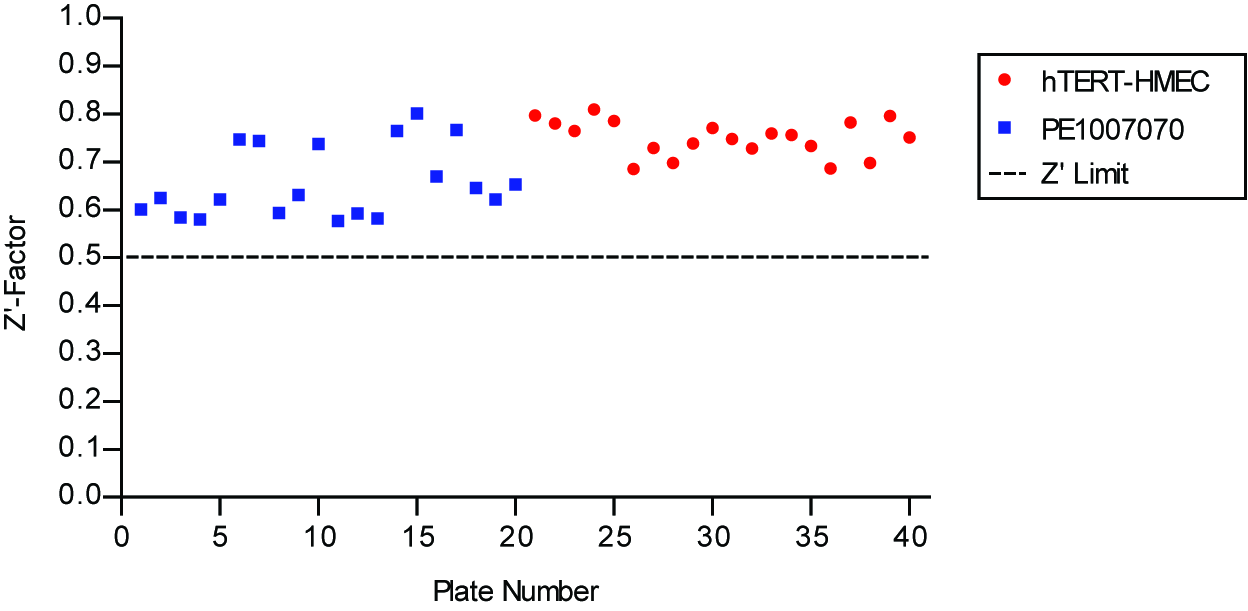

Supplement: Additional file 8 — Supplemental figure 4. The Z'-Factor for each plate was calculated using the average percent viability of the 20 μM doxorubicin wells (positive control) and 0.2% v/v DMSO wells (negative control). [file bcr3452-S8.TIFF]

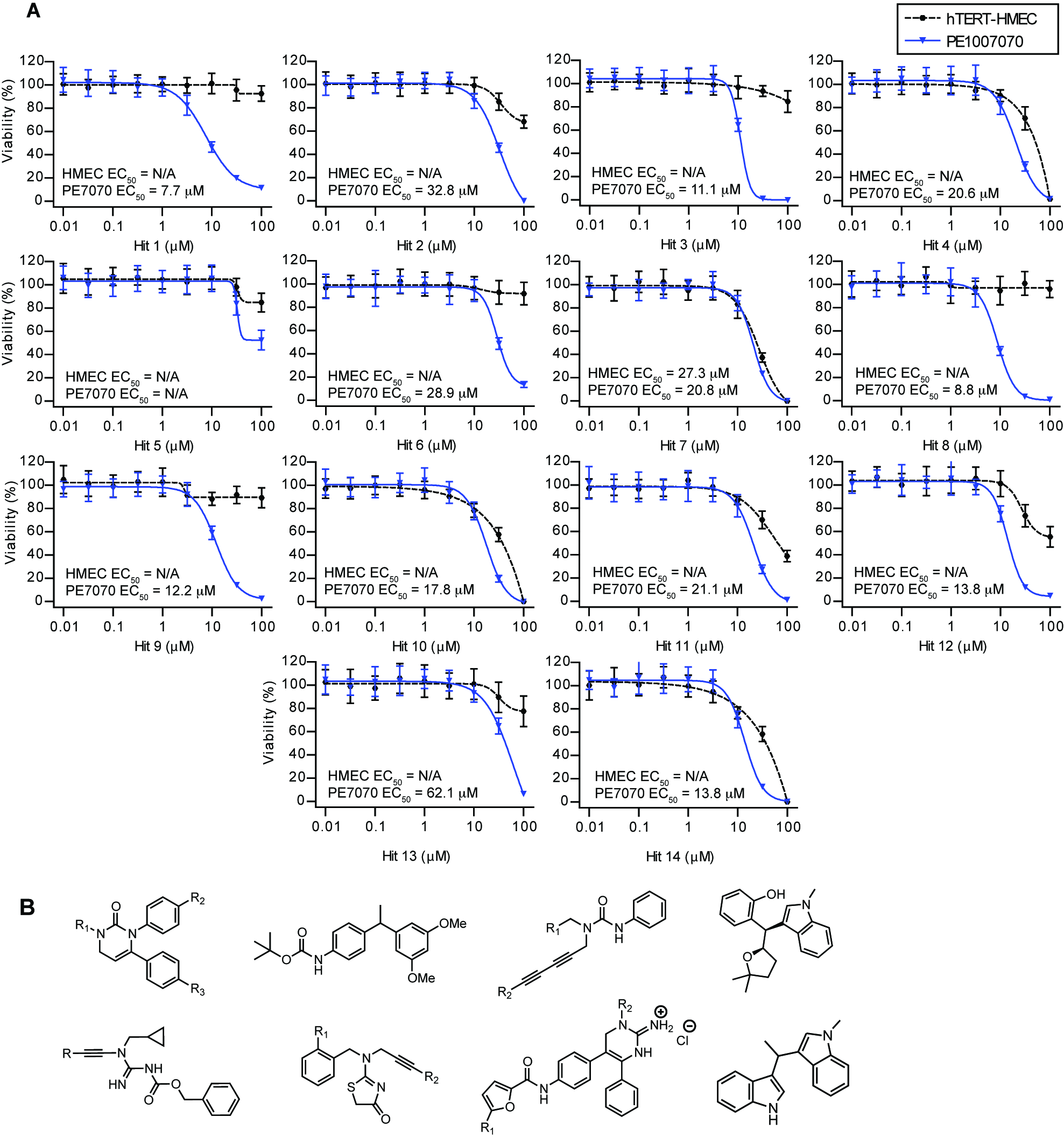

Supplement: Additional file 9 — Supplemental figure 5. (A) Dose response of the top 14 selective hits from the screen against the hTERT-HMEC and PE1007070 cells after four days of treatment. Cell viability was determined using a luciferase-based ATP viability assay, which was normalized to the untreated vehicle control. Error bars represent standard deviation. N/A denotes that data could not be fitted. (B) Representative small molecules and substructures of hits identified from the screen. [file bcr3452-S9.TIFF]

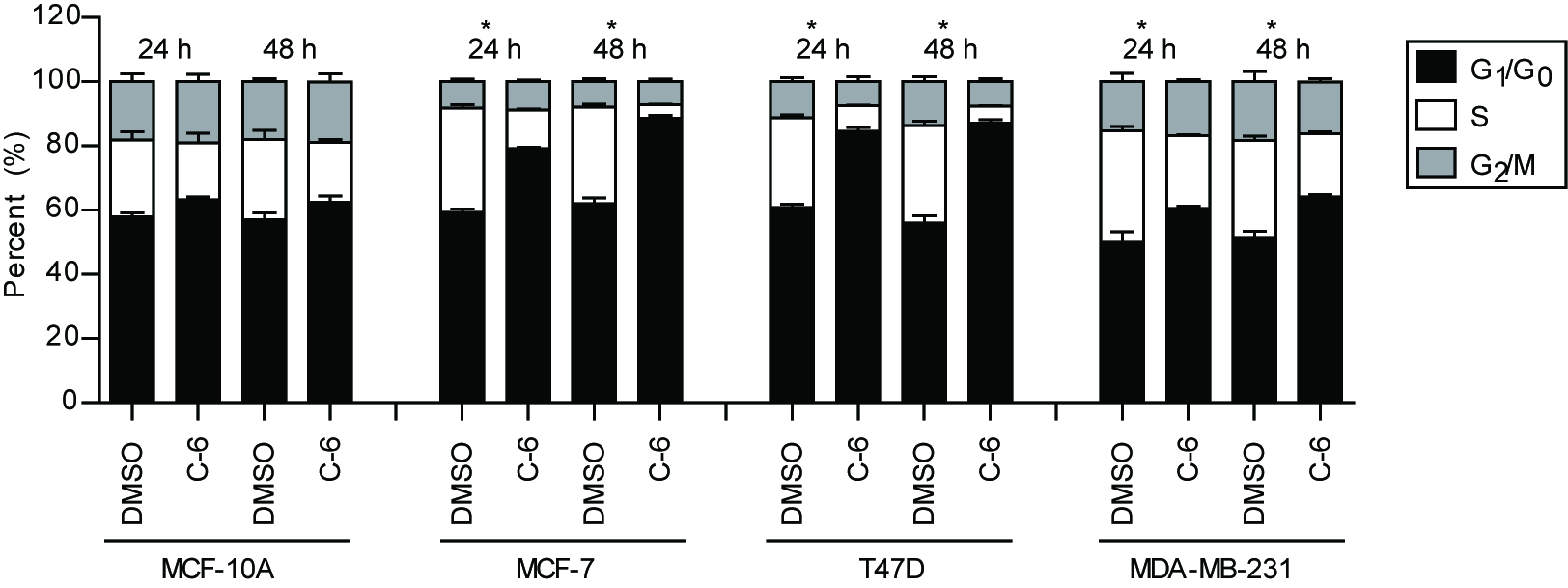

Supplement: Additional file 10 — Supplemental figure 6. MCF-10A, MCF-7, T47D, and MDA-MB-231 cells were treated with DMSO or 15 μM C-6 for 24 hours or 48 hours followed by addition of 10 μM BrdU for 30 minutes. The cells were stained for BrdU, PI and analyzed by FACS to determine the percentage of cells in the G1/G0, S, and G2/M phase. Asterisks (*) denote P-value < 0.05 of difference between percentages of cells in S phase. [file bcr3452-S10.TIFF]

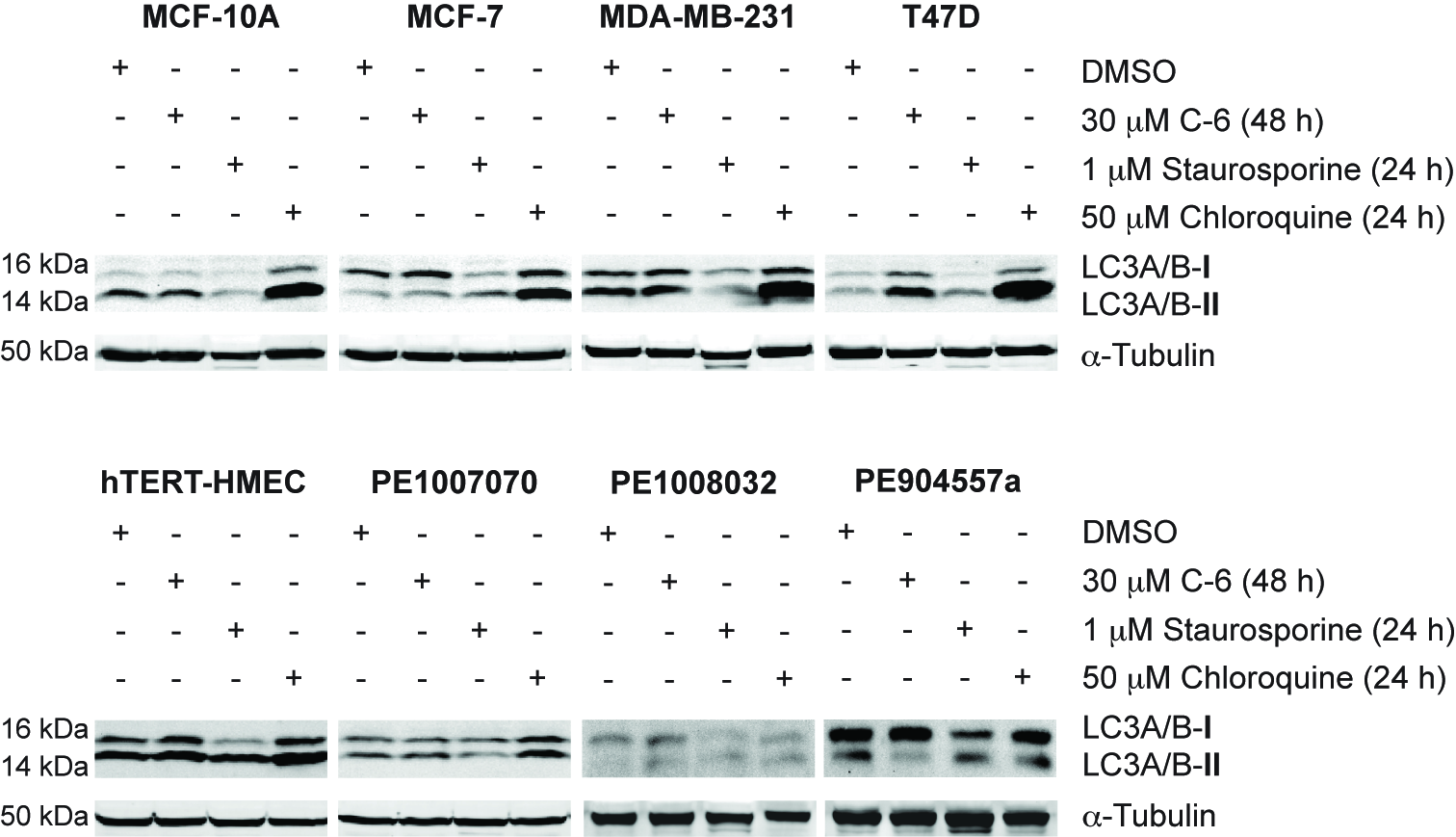

Supplement: Additional file 11 — Supplemental figure 7. C-6-induced cell death is independent of autophagy. MCF-10A, MCF-7, MDA-MB-231, T47D, hTERT-HMEC, PE1007070, PE108032 and PE904557a cells were treated with DMSO (48 hours), 30 μM C-6 (48 hours), 1 μM staurosporine (24 hours) or 50 μM chloroquine (24 hours) and resulting whole cell lysates were analyzed by Western blot for LC3A/B. [file bcr3452-S11.TIFF]
