# Supplementary material for: Development of a screen to identify selective small molecules active against patient-derived metastatic and chemoresistant breast cancer cells
Source: Breast Cancer Res. 2013 Jul 23;15(4):R58. doi: 10.1186/bcr3452 (PMC4028696; doi:10.1186/bcr3452)
Supplement: Additional file 6 — Supplemental table 4. EC50 values of chemotherapies after four days of treatment. [file bcr3452-S6.PDF]

Supplemental Table 4. EC_{50} values of chemotherapies after 4 days of treatment

Compound	MCF-7	MDA-MB-231	T47D	PE1007070	PE1008032	PE904557a
Doxorubicin	261 nM	110 nM	192 nM	677 nM	346 nM	736 nM
Taxol	0.299 nM	2.52 nM	13.7 nM	N/A[1]	N/A[1]	N/A[1]
Gemcitabine	2.73 nM	9.63 nM	6.29 nM	N/A[1]	N/A[1]	N/A[1]
17-AAG	867 nM	1.50 μM	1.41 μM	1.15 μM	1.66 μM	36.8 nM
Bortezomib	5.71 nM	35.0 nM	33.0 nM	14.6 nM	3.33 nM	32.0 nM
LBH589	21.6 nM	23.1 nM	31.0 nM	185 nM	13.7 nM	90.5 nM
Cisplatin	8.67 μM	6.88 μM	50.2 μM	10.1 μM	19.4 μM	N/A[1]

[1]Data could not be fitted.
